# Complete genome analysis of *Salmonella enterica* subsp. *enterica* serovar Typhimurium strain K79 isolated from the Buriganga river sediment in Bangladesh

**DOI:** 10.1128/mra.00600-25

**Published:** 2025-09-23

**Authors:** Durdana Mahin Priom, Spencer Mark Mondol, Nayeema Talukder Ema, Md. Rafiul Islam, Md. Mizanur Rahaman

**Affiliations:** 1 Department of Microbiology, University of Dhaka 95324, Dhaka, Bangladesh; University of Maryland School of Medicine, Baltimore, Maryland, USA

**Keywords:** Buriganga River, *Salmonella enterica*, Bangladesh, complete genome, Typhimurium

## Abstract

We report the complete genome of a *Salmonella enterica* subsp. *enterica* serovar Typhimurium strain K79, isolated from Buriganga River sediment in Bangladesh. The genome size was 48,25,269 bp having 4,897 coding sequences.

## ANNOUNCEMENT

In Bangladesh, whole-genome sequencing has emerged as a vital tool for elucidating the genomic characteristics of clinically and epidemiologically significant pathogens, contributing to improved public health surveillance (1–4). The Buriganga River, a vital waterway for Dhaka, is heavily polluted due to unchecked industrial discharge and urban waste, making it a significant reservoir of antibiotic-resistant pathogens with serious implications for public and environmental health. In this study, we have isolated a strain of *Salmonella enterica* subsp. *enterica* serovar Typhimurium from sediment near Kholamora Ghat along the Buriganga River, located at 23.7154° N latitude and 90.3609° E longitude. A sample was taken from the riverbed at a depth of 5–10 cm using an auger, then preserved in a sterile plastic bag and transported to the laboratory at 4°C in an insulated box. The sample (0.1 mL) was cultured through the spread plate method on xylose lysine deoxycholate agar plate. The single colonies were subcultured at 37°C to get a pure colony. Genomic DNA was extracted using the Genomic DNA Purification Kit for gram-negative bacteria (New England Biolabs, UK) in accordance with the manufacturer’s instructions (NEB#T3010). Whole-genome sequencing was performed on the Illumina MiniSeq platform at the Bangladesh Council of Scientific and Industrial Research (BCSIR). Paired-end libraries were prepared using the Nextera XT DNA Library Preparation Kit (Catalog: FC-131-1096), generating an average insert size of approximately 150 bp. Raw sequencing reads in FASTQ format were assessed for quality using FastQC (v0.11) (5) and subsequently trimmed using Trimmomatic (v0.39) (6) to remove low-quality bases and adapter sequences by setting parameters having a minimum average quality score of 30 and a minimum read length of 50 bp. High-quality reads were assembled *de novo* using SPAdes (v3.15.4) (7) and scaffolded through Multi-CSAR tool (8) using default parameters. Completeness of the scaffolded genome was checked using CheckM (9). The scaffolded genome was annotated using RASTtk (10) through BV-BRC (11). The circular genome was visualized using Proksee (12), and identification was performed with KmerFinder tool (13). Multi-locus sequence typing (MLST) was performed using the MLST 2.0 tool (14). Antibiotic resistance genes were investigated through CARD:RGI (15) server using default parameters. The virulence factor genes and pathogenicity were determined using the VFDB 2.0^16^) tool using default parameters.

**Fig 1:**
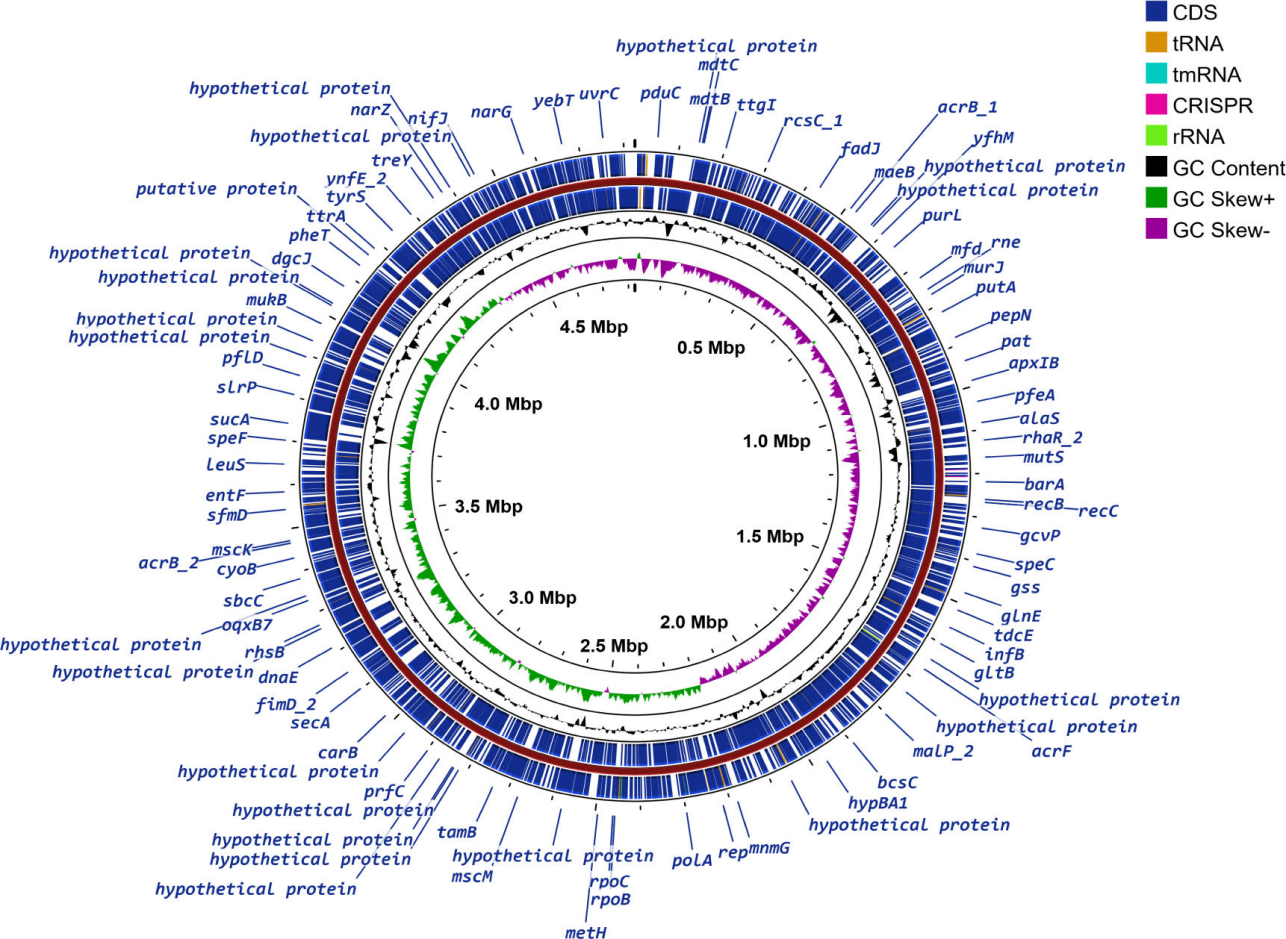
Circular genome map of *Salmonella enterica* subsp. *enterica* serovar Typhimurium strain K79 depicting the organization of CDS, tRNA, rRNA, tmRNA, GC content, and GC skew across the genome.

After sequencing, 21,60,13,400 reads were generated. The assembly coverage was 22.5×. The scaffolded complete genome was identified as a strain (K79) of *Salmonella enterica* subsp. *enterica* serovar Typhimurium having a genome size of 48,25,269 bp and 4,897 coding sequences (CDS). Genome annotation and mapping revealed key genomic features, as detailed in Table 1 and Fig. 1. The isolate had 594 hypothetical CDS, and through in-depth analysis of these hypothetical proteins, it might be possible to find out proteins having novel functions (17). *Salmonella enterica* subsp. *enterica* serovar Typhimurium K79 harbored 26 antimicrobial resistance genes. Among the genes, *aac*(6')-Iaa is notable, which is an acquired aminoglycoside resistance gene. Additionally, the strain had a 93.7% probability of being a human pathogen with 1,208 pathogenic gene families. The isolate harbored virulence factors of fimbrial adherence determinants such as Fim, Csg, Bcf, Lpf, Saf, Stb, Stc, Std, Sth, Sti, and Stj. Additionally, the isolate had genes for magnesium uptake (*mgt*B and *mgt*C), nonfimbrial adherence determinants (*mis*L, *rat*B, *shd*A, and *sin*H), and regulation (*pho*P and *pho*Q). The strain also encodes a type III secretion system within *Salmonella* pathogenicity island 1 and *Salmonella* pathogenicity island 2. This study provided insights into the genomic characteristics of a pathogenic *Salmonella enterica* strain isolated from an environmental source, highlighting the significance of adopting a “One Health” approach in the context of Bangladesh.

**TABLE 1:** General features of the annotated genome

BV-BRC (RASTtk) Annotation	*Salmonella enterica* subsp. *enterica* ser. Typhimurium K79
Completeness of genome	100 (CheckM)
Coarse consistency	99.7
Fine consistency	99.4
Contigs	1
Genome length	4,825,269
GC content	52.158756
Contig L50	1
Contig N50 (bp)	4,825,269
CDS	4,897
CDS ratio	1.0148658
Hypothetical CDS	594
tRNA	77
rRNA	7

## Data Availability

The annotated complete genome sequence has been deposited in the NCBI under the DDBJ/ENA/GenBank accession number CP169304. The raw data are available in the NCBI Sequence Read Archive under accession number SRR33800280.
